# Phenotypic and genotypic discrepancies for carbapenemase-producing *Citrobacter freundii* in multiple isolates from a single patient

**DOI:** 10.1186/s12941-023-00579-x

**Published:** 2023-04-13

**Authors:** Stephen M. Brecher, Isabella A. Tickler, Fred C. Tenover

**Affiliations:** 1 VA Boston Healthcare System, Boston, MA 02130 USA; 2 Boston University School of Medicine, Boston, MA 02118 USA; 3 Cepheid, 904 Caribbean Drive, Sunnyvale, CA 94089 USA

**Keywords:** Carbapenemase, Carbapenem resistance, *Citrobacter freundii*

## Abstract

**Background:**

Carbapenemase-producing gram-negative organisms continue to be a significant healthcare concern and a therapeutic challenge. Members of the genus *Citrobacter* have emerged as increasingly multidrug resistant and versatile healthcare-associated pathogens. In this study we investigated five KPC-producing *Citrobacter freundii* isolates, from the same patient, that presented unusual phenotypic characteristics including false susceptibility to carbapenems detection by culture-based methods.

**Methods:**

The isolates were tested for antimicrobial susceptibility using broth microdilution and disk diffusion. Production of serine carbapenemase was confirmed with the mCIM (modified carbapenem inactivation method) test. Genotypes were determined by PCR and whole genome sequencing analysis.

**Results:**

The five isolates were susceptible to meropenem by broth microdilution and presented varying colonial morphologies and levels of susceptibility to carbapenems by multiple phenotypic methods, despite being positive for carbapenemase production by mCIM and positive for *bla*_KPC_ by PCR. Whole genome sequence analysis showed that three of the five highly related isolates harbor an additional gene cassette, including *bla*_CARB-2_, *ant*(2''), *aad*A2, *dfrA*19, *catB*3, *cmlA*1, *mph*(E), *msr*(E), and *qnrA*1. The presence of these genes explains the difference in phenotypes observed.

**Conclusion:**

Failure to detect and completely eradicate the carbapenemase-producing *C. freundii* in the urine with ertapenem therapy, likely due to the presence of a heterogeneous population, resulted in the phenotypic and genotypic adaptations of the organism as it disseminated to the bloodstream and kidneys. The fact that carbapenemase-producing *C. freundii* can elude detection by phenotypic methods and can so easily acquire and transfer resistance gene cassettes is of concern.

**Supplementary Information:**

The online version contains supplementary material available at 10.1186/s12941-023-00579-x.

## Introduction

The dissemination of carbapenem-resistant gram-negative organisms continues to be a significant healthcare concern and a therapeutic challenge, especially for those organisms that are carbapenemase producers (CP) [1]. While infections caused by CP *Klebsiella pneumoniae*, *Escherichia coli*, *Pseudomonas aeruginosa* and *Acinetobacter baumannii* are frequently reported and often associated with increased morbidity and mortality [2], infections caused by CP *Citrobacter* spp., are less commonly reported. Members of the genus *Citrobacter*, which are facultatively anaerobic Gram-negative Enterobacterales, are widely distributed in the environment, have emerged as versatile healthcare-associated pathogens, and can acquire a variety of resistance genes, including carbapenemases [3, 4]. Among *Citrobacter* spp., *Citrobacter freundii* is the species most frequently associated with healthcare-associated opportunistic infections. *C. freundii* is increasingly linked to a wide spectrum of infections, including those in the urinary tract, respiratory tract, and bloodstream [5, 6]. Herein, we report a case of infection with *C. freundii,* which was isolated from a patient’s urine, blood, and kidney over the course of a few days. The isolates were all positive for the presence of *bla*_KPC_ by PCR but showed variable susceptibility to carbapenems by multiple phenotypic methods. The isolates also manifested different colony morphologies and levels of susceptibility to other antimicrobial agents. Herein we investigate the isolates, through antimicrobial susceptibility testing and whole genome sequencing, to better understand the discrepancies observed between their phenotypes and genotypes.

## Methods

### Case summary

A 34-year-old male patient was admitted to VA Boston Healthcare System (laboratory A) from an outside rehabilitation facility with a 6-month history of transverse myelitis and anti-phospholipid syndrome. According to the patient’s medical record, a multidrug-resistant *Citrobacter freundii* had been isolated from a urine specimen prior to transfer to hospital A. The isolate was resistant to cefoxitin and ceftriaxone but susceptible to imipenem. At laboratory A, the patient’s urine culture grew a multi-drug resistant *C. freundii*, which was resistant to cefoxitin, ceftazidime, cefepime, ceftriaxone, trimethoprim- sulfamethoxazole, levofloxacin, and ciprofloxacin. It was intermediate to piperacillin-tazobactam and to gentamicin and tobramycin (MicroScan Walk-Away, Beckman-Coulter, Sacramento, CA). The patient was treated with ertapenem, and a follow-up urine specimen taken one week later showed no growth. Two weeks after admission, one bottle (aerobic) of a single blood culture set (BD BACTEC FX 40, Cockeysville, MD) was positive for Gram-negative rods. An aliquot from the positive bottle was used to inoculate a BioFire Blood Culture Identification Panel Multi-Plex PCR (BCID, BioFire, Salt Lake City, Utah) and the organism was identified as a member of the Family *Enterobacterales* that was positive for *bla*_KPC_. The organism was identified using the MicroScan Gram-Negative Combo 87 plate as *C. freundii* and confirmed using MALDI-TOF (Bruker, Billerica, MA). Treatment with meropenem-vaborbactam (5 weeks) and amikacin (2 weeks followed by 6 weeks) resulted in clinical cure at all infected sites. Three isolates from blood culture, designated as BC-1 and BC-2 (two different morphotypes collected on day 1) and BC-3 (collected on day 2), and two from kidney abscess, K-1 and K-2 (collected on day 1 and day 3, respectively), were sent to Cepheid (laboratory B) for further investigation.

### Antimicrobial Susceptibility Testing (AST)

The five isolates were tested for antimicrobial susceptibility using the broth microdilution method with MicroScan Neg Detect 2 and NM56 panels (Beckman Coulter, Sacramento, CA) and with the disk diffusion method for susceptibility to 12 antimicrobial agents (ertapenem, imipenem, meropenem, cefotaxime, cefotaxime w/clavulanic acid, ceftazidime, ceftazidime w/clavulanic acid, aztreonam, cefepime, ceftriaxone, ceftazidime/avibactam, and cefiderocol). Organism identification was confirmed using MALDI-TOF (Bruker, Billerica, MA). The isolates were also tested for carbapenemase production using the modified carbapenem inactivation method (mCIM) and with the addition of ethylene diamine tetra acetic acid (eCIM). All tests were performed and interpreted according to CLSI guidelines [7]. A meropenem disk was placed on each agar plate used for subculture and colonies close to the disk were picked each time to maintain antimicrobial selection.

### Genome sequencing and analysis

Genomic DNA was extracted from pure cultures of organisms grown overnight on blood agar plates using the Qiagen DNeasy Blood and tissue kit (Qiagen, USA). Sequencing libraries were prepared using Illumina DNA Prep Kit (Illumina, USA) and sequencing was carried out on the MiSeq instrument using Reagent Kit v2 chemistry (Illumina, USA). De novo assemblies, identification using k-mer spectra, and detection of antimicrobial resistance genes were performed with the CLC Genomics Workbench 21.0.5 and CLC Microbial Genomics Module 21.1 (CLCbio, Denmark). Acquired resistance genes were detected from assembled genomes using the ResFinder database (downloaded on 2022/07/26) with 80% minimum identity and 60% minimum length [8]. Plasmids were identified from the assembled genomes using the Center for Genomic Epidemiology tools PlasmidFinder 2.1 and Mobile Element Finder with thresholds set at 95% minimum identity and 60% minimum coverage (http://www.genomicepidemiology.org/) [9]. Alignments were analyzed and viewed with the SnapGene software version 5.1.7 (GSL Biotech, snapgene.com). This Whole Genome Shotgun project was deposited at DDBJ/ENA/GenBank under BioProject PRJNA851423.

## Results

### Antimicrobial susceptibility testing

Representative antimicrobial agent broth microdilution (BMD) results for the *C. freundii* isolates from the urine, blood, and kidney cultures at both laboratories are shown in Table 1. Results obtained by DD, mCIM/eCIM, and BMD are summarized in Table 1 and Additional file 2: Tables S1 and S2. All isolates were positive for production of serine carbapenemase by mCIM/eCIM.

**Table 1:** MICs of antimicrobial agents for isolates from multiple body site, mCIM/eCIM interpretations and PCR results

Antimicrobials	Samples				
	BC-1	BC-2	BC-3	K-1	K-2
Amikacin (µg/ml)	≤ 16 (S)	≤ 16 (S)	≤ 16 (S)	≤ 16 (S)	≤ 16 (S)
Aztreonam (µg/ml)	≤ 4 (S)	≤ 4 (S)	> 16 (R)	> 16 (R)	> 16 (R)
Cefepime (µg/ml)	≤ 2 (S)	≤ 2 (S)	8 (SDD)	> 16 (R)	> 16 (R)
Cefotaxime (µg/ml)	8 (R)	≤ 2 (S)	> 32 (R)	> 32 (R)	> 32 (R)
Ceftazidime (µg/ml)	4 (S)	4 (S)	16 (R)	> 16 (R)	16 (R)
Ceftazidime/Avibactam (µg/ml)	≤ 4 (S)	≤ 4 (S)	≤ 4 (S)	≤ 4 (S)	≤ 4 (S)
Ceftolozane/Tazobactam (µg/ml)	≤ 2 (S)	≤ 2 (S)	8 (R)	8 (R)	8 (R)
Ceftriaxone (µg/ml)	8 (R)	2 (I)	8 (R)	> 32 (R)	> 32 (R)
Ciprofloxacin (µg/ml)	1 (R)	1 (R)	> 2 (R)	> 2 (R)	> 2 (R)
Ertapenem (µg/ml)	≤ 0.5 (S)	≤ 0.5 (S)	1 (I)	1 (I)	≤ 0.5 (S)
Gentamicin (µg/ml)	≤ 2 (S)	≤ 2 (S)	8 (I)	> 8 (R)	4 (S)
Imipenem (µg/ml)	2 (I)	2 (I)	2 (I)	2 (I)	2 (I)
Levofloxacin (µg/ml)	≤ 0.5 (S)	≤ 0.5 (S)	> 4 (R)	> 4 (R)	> 4 (R)
Meropenem (µg/ml)	≤ 1 (S)	≤ 1 (S)	≤ 1 (S)	≤ 1 (S)	≤ 1 (S)
Meropenem/Vaborbactam (µg/ml)	≤ 2 (S)	≤ 2 (S)	≤ 2 (S)	≤ 2 (S)	≤ 2 (S)
Moxifloxacin (µg/ml)	≤ 2 (S)	≤ 2 (S)	> 4 (R)	> 4 (R)	> 4 (R)
Piperacillin/Tazobactam (µg/ml)	≤ 8 (S)	≤ 8 (S)	32 (R)	64 (R)	64 (R)
Tobramycin (µg/ml)	≤ 2 (S)	≤ 2 (S)	8 (I)	> 8 (R)	8 (I)
mCIM/eCIM interpretation	Serine carbapenemase detected	Serine carbapenemase detected	Serine carbapenemase detected	Serine carbapenemase detected	Serine carbapenemase detected
Carbapenemase gene detected ^a^	*bla*_KPC_	*bla*_KPC_	*bla*_KPC_	*bla*_KPC_	*bla*_KPC_

MICs in µg/ml and categorical interpretation (in parentheses)

Interpretation of MICs from CLSI M100 S32 2022

*S* Susceptible; *I* Intermediate; *SDD* Susceptible Dose Dependent; *R* Resistant

^a^KPC target identified on the Xpert Carba-R assay (Cepheid)

### Broth microdilution (BMD)

All isolates were susceptible to meropenem and intermediate to imipenem by the BMD method (Table 1). MICs of ertapenem and meropenem for the blood culture isolates of *C. freundii* from hospital A were identical to the ertapenem and meropenem MICs of the original urine isolate (susceptible to both drugs) (Additional file 2: Table S1). BC-1 and BC-2, however, had an extended MIC profile suggestive of organisms with a non-induced *ampC,* i.e., they were susceptible to ceftazidime and cefepime, had variable levels of resistance (ranging from susceptible to resistant) to ceftriaxone, and were susceptible to piperacillin/tazobactam. Isolate BC-3, collected a day later, had a resistance profile closer to that of the urine isolate, i.e., it was resistant to ceftazidime, ceftriaxone, cefepime [one laboratory reported susceptible dose-dependent (SDD)] and resistant to piperacillin/tazobactam. The patient had also developed a kidney abscess and multiple drainage cultures again revealed *C. freundii* with different morphologies and resistance profiles. The isolates from kidney abscess, K-1 and K-2, presented a resistance profile similar to that of the original urine isolate and BC-3. The AST profiles were intermediate to imipenem. Both isolates were susceptible to ertapenem, although in laboratory B, K-1 was intermediate to ertapenem. All the isolates were resistant to 1st and 2nd generation cephalosporins but showed varying levels of susceptibility to 3rd generation cephalosporins. Isolates BC-1 and BC-2 were susceptible to the 4th generation cephalosporin, cefepime, while the remaining isolates were resistant with one reported as cefepime SDD (Table 1). Variable resistance to aztreonam was also observed among the isolates, with BC-1 and BC-2 showing susceptibility and BC-3, K-1, and showing K-2 resistance. All the isolates were resistant to amoxicillin/ clavulanic acid and ampicillin/sulbactam; however, susceptibilities to ceftolozane-tazobactam and piperacillin-tazobactam were variable. All five isolates were susceptible to ceftazidime/avibactam, meropenem/vaborbactam, and amikacin, although BC-3, K-1 and K-2 displayed varying degrees of resistance to the other aminoglycosides (Table 1). Variable susceptibility patterns were also observed with fluoroquinolones with resistance to ciprofloxacin for all isolates, but with BC-1 and BC-2 showing susceptibility to moxifloxacin and levofloxacin, while BC-3, K-1 and K-2 were resistant to both agents.

### Disk diffusion (DD)

The variable resistance profiles to the carbapenems observed with BMD were more evident among the DD results. BC-1 and BC-2 were susceptible to ertapenem and meropenem and intermediate to imipenem, while BC-3, K-1 and K-2 were either intermediate or resistant to the carbapenems (Additional file 2: Table S2). The same resistance patterns were found among cephalosporins and aztreonam.

### Sequencing analysis: antibiotic resistance genes and plasmids

**Fig. 1:**
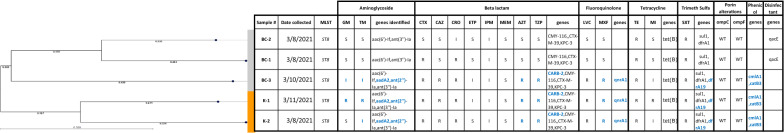
Selected antimicrobial susceptibility results (broth microdilution) of the five isolates by antimicrobial class with the genes identified by sequencing analysis (ResFinder database). A neighbor-joining (NJ) phylogenetic tree compares the isolates based on whole genome alignment percent. Abbreviations: gentamicin (GM), tobramycin (TM), cefotaxime (CTX), ceftazidime (CAZ), ertapenem (ETP), imipenem (IPM), meropenem (MEM), aztreonam (ATM), piperacillin-tazobactam (TZP), levofloxacin (LVX), moxifloxacin (MXF), tetracycline (TE), minocycline (MI), trimethoprim-sulfamethoxazole (SXT), wildtype (WT)

**Fig. 2:**
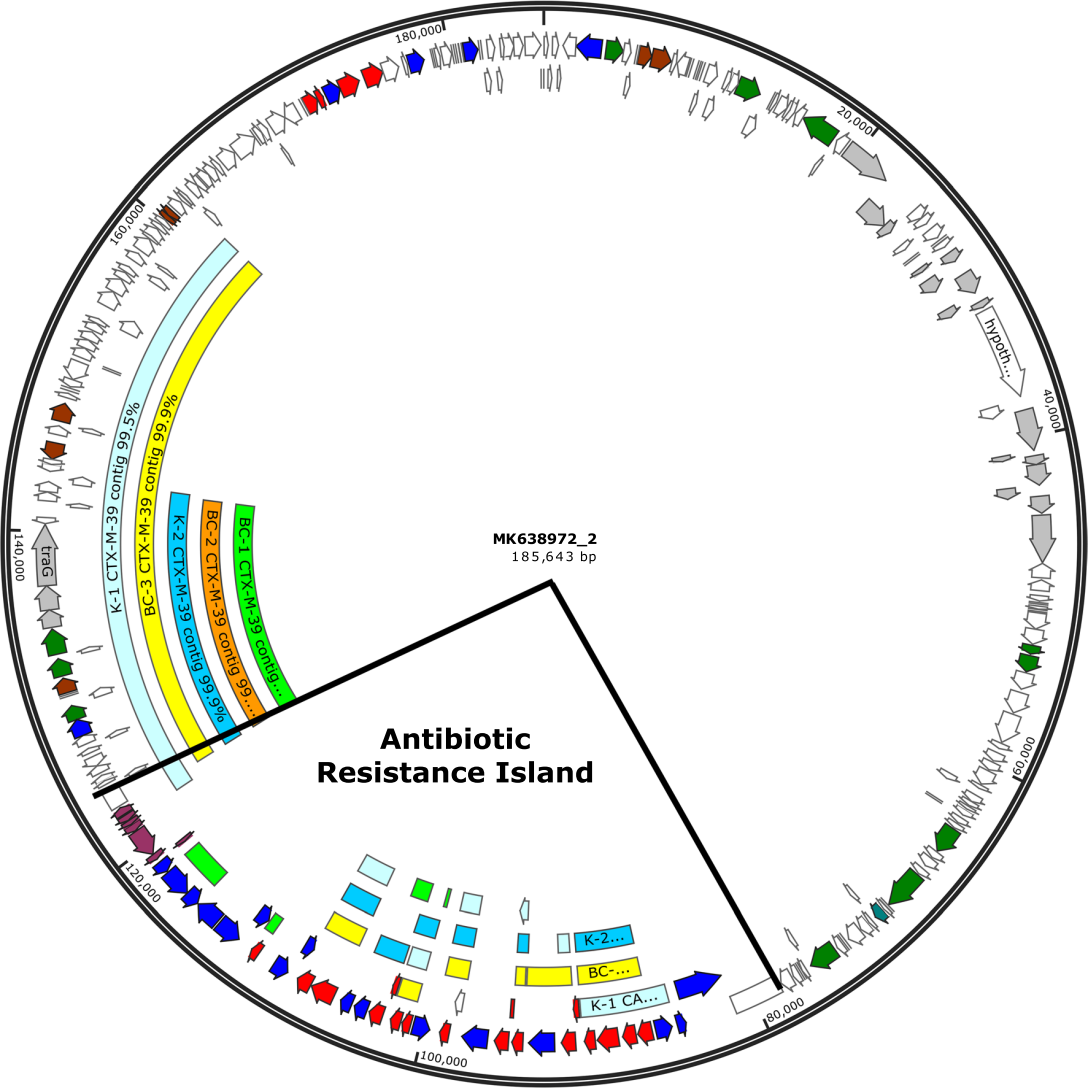
SnapGene alignment of the contigs of the five isolates harboring the resistance genes *bla*_*CARB-2*_*, bla*_CTX-M-39_*, aadA2, ant(2'')-Ia, dfrA19, cmlA1,catB3, mph(E), msr(E), qacE, sul1,* and *qnrA1* with plasmid pMG252 described in *E. coli* strain J53 (Accession MK638972). Contigs are color-coded by isolate: BC-1 green, BC-2 orange, BC-3 yellow, K-1 light blue, K-2 dark blue. The contigs carrying *bla*_CTX-M-39_ aligned with plasmid pMG252, except for the gene itself which was not present on pMG252. Genes are color-coded according to the following key: dark blue, transposase and integrase genes; red, antimicrobial resistance genes; dark red, mercury resistance genes; grey, *tra* gene complex; dark green, DNA metabolism; brown, plasmid maintenance and replication; white, all other genes

As expected, the isolates were highly related, with an alignment percentage between 98.99% and 99.40%. Figure 1 groups the five isolates by alignment percentage, relative to type of sample and genotypic and phenotypic results (Fig. 1). All five *C. freundii* isolates were sequence type (ST)-8 by the *Citrobacter* spp. PubMLST 7-loci scheme present in CLC Genomic Workbench (CLC Type with MLST scheme 1.3) [10] and each harbored the *bla*_KPC-3_ carbapenemase gene, the chromosomal *bla*_CMY-116_ *ampC* beta-lactamase gene, and the *bla*_CTX-M-39_ extended-spectrum beta-lactamase (ESBL) gene. All five isolates also shared aminoglycoside [*aac*(6’)-If], tetracycline (*tet*B), aminocyclitol resistance genes [*ant*(3″)-Ia], and folate pathway antagonist genes (*dfr*A1, *sul*1), which were likely chromosomal (Fig. 1). However, isolates BC-3, K-1, and K-2 harbored additional resistance genes, including the class A carbenicillin-hydrolyzing beta-lactamase, *bla*_CARB-2_, the *ant*(2'') and *aad*A2 aminoglycoside resistance genes, *dfrA*19, which produces a trimethoprim-resistant dihydrofolate reductase, the *catB*3 and *cmlA*1chloramphenicol resistance determinants, the *mph*(E) and *msr*(E) macrolide resistance genes, and *qnrA*1, which produces a quinolone resistance pentapeptide repeat protein. Isolates BC-1 and BC-2, in turn, carried the *qacE* gene, which confers resistance to quaternary ammonium compounds. No alterations of the porin genes *ompC* and *ompF* were found. PlasmidFinder identified the same two plasmids in all five isolates, namely, IncC (Sequence Type 3 by pMLST 2.0 Server) and pKPC-CAV1321. A BLAST (Basic Local Alignment Search tool, National Library of Medicine) search of the genomic region encompassing the *bla*_CARB-2_ gene of isolates BC-3, K-1 and K-2 returned 100% identity to a portion of the antimicrobial resistance island of plasmid pMG252, described in an *Escherichia coli* isolate with elevated quinolone resistance [11]. The contigs containing resistance genes *bla*_CTX-M-39_, *aad*A2, *ant*(2'')-Ia, *dfrA*19, *cml*A1, *catB*3, *mph*(E), *msr*(E), *qac*E, *sul*1, *and qnrA*1 also shared 99–100% identity with regions of plasmid pMG252, although only the genomic environment surrounding *bla*_CTX-M-39_ was found on the plasmid, but not the actual *bla*_CTX-M-39_. All regions showing similarity with plasmid pMG252 are shown in Fig. 2. A BLAST search of the *bla*_KPC-3_-containing contigs returned only partial matches to several published *Citrobacter* spp. plasmids. The ~ 10,000 bp region surrounding the *bla*_KPC-3_ gene, however, returned a 99–100% match and 100% coverage to published sequences of a *bla*_KPC-2 or KPC-3_-carrying transposon Tn*4401* integrated within a Tn*2*-like element. This transposon is also found on plasmid pKPC_CAV1321 [12], originally identified by CGE PlasmidFinder. Additional file 1: Fig. S1 illustrates the alignment of the five *bla*_KPC-3_ genomic environments with the Tn*4401*b-1 transposon of pKPC_CAV1321-45. The only mismatch is due to the fact that pKPC_CAV1312-45 harbors a *bla*_KPC-2_ instead of *bla*_KPC-3._

## Discussion

We confirmed that the five *C. freundii* isolates, although highly related, differed by several resistance determinants, consistent with the varying phenotypes observed among the isolates throughout the study. All isolates carried *bla*_KPC-3_ but were phenotypically susceptible or intermediate to meropenem. Carbapenem heteroresistance and the challenge of its detection by phenotypic methods, particularly automated systems, has been previously reported for *bla*_KPC_-producing *Klebsiella pneumoniae* and other Gram-negative rods [13, 14]. This would explain why in our study the disk diffusion method more accurately detected the carbapenem-resistant subpopulations than broth microdilution. Arana et al., who studied the molecular epidemiology of CP *Citrobacter* spp. in Spain, described a similar phenomenon, reporting that 12.7% of the CP *Citrobacter* spp. were carbapenem-susceptible using EUCAST interpretive criteria, although half of them were intermediate to imipenem by CLSI breakpoints [4]. The three isolates harboring the *bla*_CARB-2_ gene presented a different resistance profile from the other two, which included reduced susceptibility to ceftazidime, cefepime, aztreonam and piperacillin-tazobactam. Conjugation experiments performed on *E. coli* by Lin et al. demonstrated that carriage of the closely related *bla*_CARB-3_ gene was associated with reduced susceptibility to piperacillin-tazobactam, ceftazidime, and cefepime [15]. The additional resistance genes found among our isolates were located in gene cassettes within class 1 integrons, demonstrating that *C. freundii* can acquire resistance genes from other organisms. The acquisition of the gene cassette by isolates BC-3, K-1 and K-2 conferred increased resistance to four classes of antimicrobial agents, including aminoglycosides, beta-lactams, fluoroquinolones, and phenicols. Gene cassettes on Class 1 integrons have been reported frequently among multi-drug resistant *Citrobacter* spp. [16]. We were unable to sequence the original urine isolate, but from its antimicrobial resistance profile, we can assume that it harbored the gene cassette that included *bla*_CARB-2_, and likely the rest of the cassette: the *ant*(2''), *aad*A2, *dfrA*19, *catB*3, *mph*(E), *msr*(E), and *qnrA*1, suggesting a shuffling and/or excision of resistance genes during the progression of the infection from kidneys to bloodstream.

## Conclusions

We conclude that based on false susceptible phenotypic carbapenem susceptibility results, inappropriate antimicrobial therapy resulted in dissemination of the *Citrobacter freundii* isolate from the urine to the kidneys and to the blood. Clearance of the organism was achieved only when appropriate antimicrobial therapy was administered. The fact that carbapenemase-producing *C. freundii* can elude detection by phenotypic methods and can so easily acquire and transfer resistance gene cassettes is a challenge for the clinical microbiology laboratory.

Laboratories should consider testing isolates of *C. freundii* from serious systemic infections both phenotypically and genotypically for carbapenemase production and carbapenemase gene carriage, respectively, to ensure detection of stealth resistance profiles.

## Supplementary Information

**Additional file 1. ** SnapGene alignment of the contigs of the five isolates harboring the blaKPC-3 gene with plasmid pKPC_CAV1312-45 (CP011608).

**Additional file 2.** **Table S1**. Antimicrobial susceptibility testing results (broth microdilution) by laboratory site. **Table S2**. Disk diffusion and mCIM/eCIM results. **Table S3**. Plasmids and resistance genes identified by WGS.

## Data Availability

All data generated during this study are included in this published article and its supplementary information files. The Whole Genome Shotgun project was deposited at DDBJ/ENA/GenBank under BioProject PRJNA851423 (https://www.ncbi.nlm.nih.gov/bioproject/PRJNA851423).
