# Supplementary material for: Phenotypic and genotypic discrepancies for carbapenemase-producing Citrobacter freundii in multiple isolates from a single patient
Source: Ann Clin Microbiol Antimicrob. 2023 Apr 13;22:24. doi: 10.1186/s12941-023-00579-x (PMC10103531; doi:10.1186/s12941-023-00579-x)
Supplement: Supplementary file 1 — Additional file 1. SnapGene alignment of the contigs of the five isolates harboring the blaKPC-3 gene with plasmid pKPC_CAV1312-45 (CP011608). [file 12941_2023_579_MOESM1_ESM.pdf]

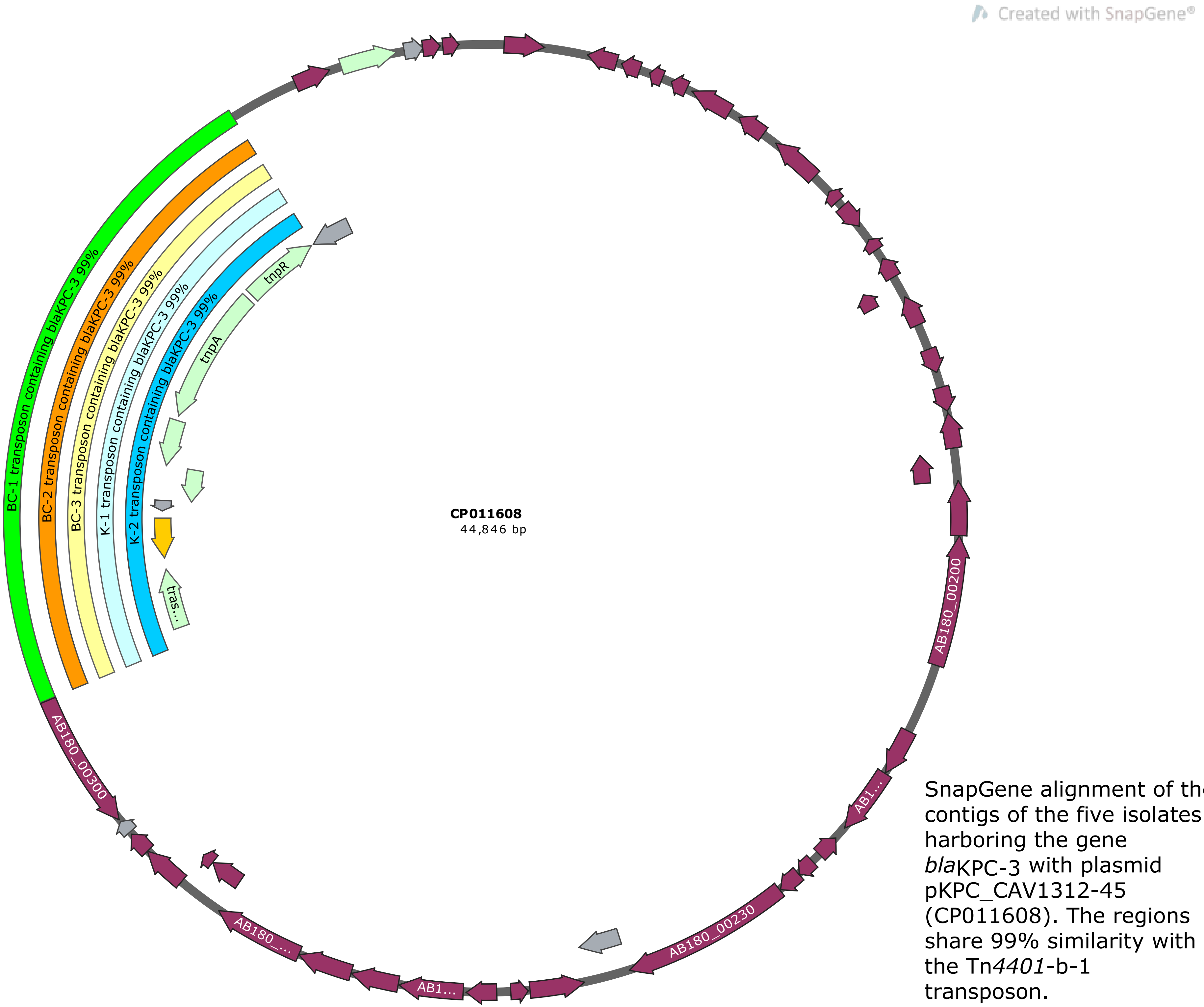

SnapGene alignment of the contigs of the five isolates harboring the gene *bla*KPC-3 with plasmid pKPC_CAV1312-45 (CP011608). The regions share 99% similarity with the Tn*4401*-b-1 transposon.
